# Gender variations in neonatal and early infant mortality in India and Pakistan: a secondary analysis from the Global Network Maternal Newborn Health Registry

**DOI:** 10.1186/s12978-020-01028-0

**Published:** 2020-12-17

**Authors:** Zubair H. Aghai, Shivaprasad S. Goudar, Archana Patel, Sarah Saleem, Sangappa M. Dhaded, Avinash Kavi, Parth Lalakia, Farnaz Naqvi, Patricia L. Hibberd, Elizabeth M. McClure, Tracy L. Nolen, Pooja Iyer, Robert L. Goldenberg, Richard J. Derman

**Affiliations:** 1 Thomas Jefferson University, Philadelphia, PA USA; 2 Women’s and Children’s Health Research Unit, KLE Academy of Higher Education and Research’s J N Medical College, Belagavi, Karnataka 590010 India; 3 Lata Medical Research Foundation, Nagpur, India; 4 Aga Khan University, Karachi, Pakistan; 5 School of Public Health, Boston University, Boston, MA USA; 6 RTI International, Durham, NC USA; 7 Department of Obstetrics and Gynecology, Columbia University School of Medicine, New York, NY USA

**Keywords:** Early neonatal mortality, Late neonatal mortality, Stillbirth, Low-middle income countries, Sex variation in mortality, Global network

## Abstract

**Background:**

To determine the gender differences in neonatal mortality, stillbirths, and perinatal mortality in south Asia using the Global Network data from the Maternal Newborn Health Registry.

**Methods:**

This study is a secondary analysis of prospectively collected data from the three south Asian sites of the Global Network. The maternal and neonatal demographic, clinical characteristics, rates of stillbirths, early neonatal mortality (1–7 days), late neonatal mortality (8–28 days), mortality between 29–42 days and the number of infants hospitalized after birth were compared between the male and female infants.

**Results:**

Between 2010 and 2018, 297,509 births [154,790 males (52.03%) and 142,719 females (47.97%)] from two Indian sites and one Pakistani site were included in the analysis [288,859 live births (97.1%) and 8,648 stillbirths (2.9%)]. The neonatal mortality rate was significantly higher in male infants (33.2/1,000 live births) compared to their female counterparts (27.4/1,000, p < 0.001). The rates of stillbirths (31.0 vs. 26.9/1000 births) and early neonatal mortality (27.1 vs 21.6/1000 live births) were also higher in males. However, there were no significant differences in late neonatal mortality (6.3 vs. 5.9/1000 live births) and mortality between 29–42 days (2.1 vs. 1.9/1000 live births) between the two groups. More male infants were hospitalized within 42 days after birth (1.8/1000 vs. 1.3/1000 live births, p < 0.001) than females.

**Conclusion:**

The risks of stillbirths, and early neonatal mortality were higher among male infants than their female counterparts. However, there was no gender difference in mortality after 7 days of age. Our results highlight the importance of stratifying neonatal mortality into early and late neonatal period to better understand the impact of gender on neonatal mortality. The information from this study will help in developing strategies and identifying measures that can reduce differences in sex-specific mortality.

## Background

The risk of mortality and morbidity has been found to be higher in male subjects compared to females during the perinatal period, infancy and childhood [1–4]. The risk of prematurity, intrauterine growth restriction and respiratory morbidities are also higher in male infants [2, 4–7]. Male infants are also at an increased risk for respiratory and gastrointestinal infections likely due to high testosterone levels that suppress the immune system [8, 9]. In high-income nations, boys are at a greater risk of neonatal and infant mortality than girls [1, 3–5]. However, several recent studies have reported higher neonatal and infant mortality in females compared to males in south Asia [10–12]. A few studies from south Asia also reported that girls experience a higher risk of late neonatal mortality (between 8 and 28 days) [6, 12, 13]. Gender preference and differential health care-seeking behavior can contribute to higher late neonatal and infant mortality in south Asian girls [12–15]. The information on gender difference in stillbirths and mortality in different periods of infancy is important for developing strategies and identifying measures directed at reducing sex-specific mortality [12]. The objective of the current study was to determine the gender differences in stillbirths, early and late neonatal mortality, and 29–42 day mortality in south Asia using Global Network data from the Maternal Newborn Health Registry (MNHR). We hypothesize that gender differences exist in fetal and neonatal survival with sex-specific trends in stillbirths, early and late neonatal mortality, and 29–42 day mortality. Understanding the variation in sex-specific differences in stillbirth and neonatal mortality will help to develop strategies to address them.

## Methods

Pregnant women included in this analysis were screened, consented and enrolled in the MNHR between January 2010 and December 2018. The Global Network’s MNHR is a prospective observational study that includes all pregnant women and delivered infants and their outcomes in defined geographic communities (clusters). The MNHR, supported by the *Eunice Kennedy Shriver* National Institute of Child Health and Human Development’s (NICHD’s) Global Network, is a multi-site research network representing partnerships between the US institutions and international investigators at study sites in India (2 sites: Nagpur and Belagavi), Pakistan, Bangladesh, Guatemala, Kenya, Democratic Republic of the Congo and Zambia. For this study, sites in India (Belagavi and Nagpur) and Pakistan (Thatta) were included. During the time of the study, the included sites had between 20 and 24 study clusters, which are defined geographic areas with approximately 300 – 500 annual births [16, 17].

The registry administrators (RAs) identify and screen all pregnant women in the study communities early in the pregnancy and prospectively follow them and their infants up to 42 days postpartum. At enrollment, basic demographic information was recorded, and follow-up visits were conducted, one within 48 h of the delivery to obtain birth outcomes and the other at 42 days postpartum to record the health status of mother and baby, as described in detail elsewhere [16, 17]. The study outcome data were based on medical record review, as well as interviews with birth attendants, the mother and the family. In addition to the prospective enrollment of pregnant women, several measures were taken to ensure accuracy of the stillbirth, maternal, and neonatal mortality data, including: supervisory oversight of the RAs’ data collection, review of the ratio of stillbirths to early neonatal deaths to identify any potential biases, and training and review of standardized definitions.

### Study outcomes and definitions

The outcomes of interest were rates of stillbirths, early neonatal mortality, late neonatal mortality, mortality between 29 and 42 days, and the number of infants hospitalized after birth.

- Stillbirth: Fetal deaths occurring at ≥ 20 weeks gestation (or for those without gestational age available ≥ 500 g).
- Neonatal mortality rate: The number of deaths during the first 28 days of life (1–28 days) per 1000 live births.
- Early neonatal mortality: The number of deaths during the first 7 days of life (1–7 days) per 1000 live births.
- Late neonatal mortality: The number of deaths between days 8–28 of life per 1000 live births.
- Post neonatal mortality: The number of deaths between days 29–42 days per 1000 live births.
- Conditions requiring hospitalization in follow-up: The number of infants admitted to the hospital after delivery and by 42 days of life.

### Data analyses

A team at each research site supervised local data collection and provided the initial review of the data. Next, the data were entered at each study site and transmitted through a secure process to the central data coordinating center, RTI International (Research Triangle Park, NC). Descriptive analyses were performed and demographic and clinical characteristics where compared for male and female genders using a Cochran–Mantel–Haenszel test stratified by cluster for categorical characteristics and a Wilcoxon rank sum test for continuous characteristics. Relative risks and 95% confidence intervals were obtained from log binomial models as a function of gender for each fetal/neonatal outcome using generalized estimating equations to account for the correlation of outcomes within cluster. All data analyses were done with SAS software v.9.4 (Cary, NC). A two-sided p-value < 0.05 was considered to be statistically significant.

### Ethics approval

Each research site obtained approval by the local ethics review committees (Aga Khan University; KLE University’s Jawaharlal Nehru Medical College, Belagavi, India; Lata Medical Research Foundation, Nagpur, India), the institutional review boards of partner U.S. universities and the data coordinating center (RTI International). All pregnant women included in the MNHR provided informed consent for participation in the study.

## Results

**Fig. 1:**
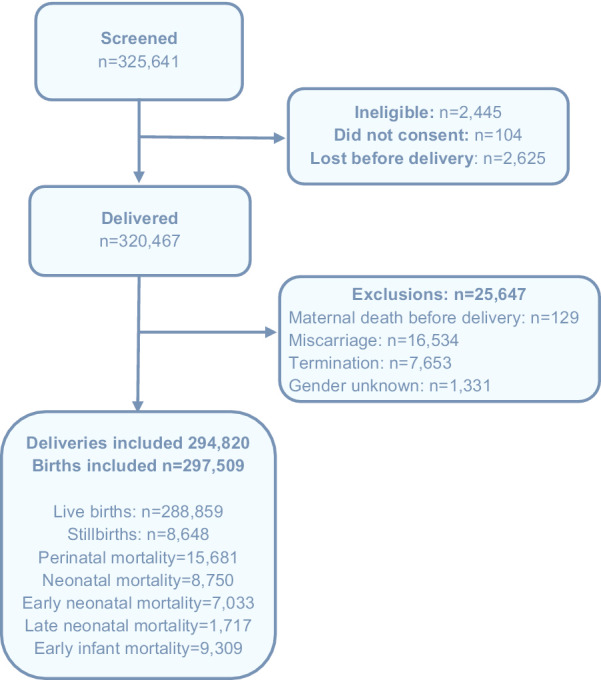
Flow diagram

A total of 325,641 women were screened between January 2010 and December 2018 at two sites in India (Nagpur and Belagavi, India) and one site in Pakistan. Most of the screened women (n = 320,467, 98.4%) were eligible, consented and delivered (Fig. 1). After excluding women with miscarriages, medical terminations of pregnancy, maternal deaths prior to delivery, and infants with gender unknown (1345), 297,509 births were included in the analysis. This included 288,859 live births (97.1%) and 8,648 stillbirths (2.9%).

The baseline demographic and clinical characteristics by infant gender are depicted in Table 1. A total of 154,790 infants (52.03%) were male and 142,719 infants (47.97%) were female, with a male to female ratio of 1,085:1,000. The sex ratio for live birth was 1,080:1,000 (149,984 males, 138,875 females), while the sex ratio for stillbirth was higher at 1,251:1,000 (4806 males, 3842 females). The differences in demographic and clinical characteristics were either not significant (maternal age, maternal education, parity, and number of antenatal care visits, hypertensive disease/severe pre-eclampsia/eclampsia, body mass index (BMI) ≥ 25 kg/m^2^, hemoglobin (Hb) < 8 g/dl, multiple birth, preterm birth and term birth) or observed difference was very small and may not be clinically significant (antepartum hemorrhage; 1.5% vs. 1.4%) [18, 19]. The demographic and clinical characteristics between the two groups were also compared by the sites (Table 2). There was a small, clinically insignificant difference in the percent of women delivering male and female infants starting prenatal care in the first trimester (75.4% vs. 76.0%), and multiple births (1.5% vs. 1.6%) at the Indian sites and severe antepartum hemorrhage at the Pakistani site (3.7% vs. 3.3%).

**Table 1:** Baseline demographic and clinical characteristics by infant gender

Characteristic	Overall	Infant Gender		p-value
		Male	Female	
Infants (%)^1^	297,509	154,790 (52.03)	142,719 (47.97)	
Maternal age, N (%)^2,3^	297,229	154,622	142,607	0.1934
< 20	18,151 (6.1)	9325 (6.0)	8,826 (6.2)	
20—35	273,435 (92.0)	142,340 (92.1)	131,095 (91.9)	
> 35	5643 (1.9)	2957 (1.9)	2686 (1.9)	
Maternal education, N (%)^2,3^	296,599	154,306	142,293	0.2556
No formal schooling	100,006 (33.7)	52,010 (33.7)	47,996 (33.7)	
Primary or Secondary	168,503 (56.8)	87,759 (56.9)	80,744 (56.7)	
University+	28,090 (9.5)	14,537 (9.4)	13,553 (9.5)	
Parity, N (%)^2,3^	294,655	153,310	141,345	0.6322
0	107,334 (36.4)	55,783 (36.4)	51,551 (36.5)	
1–2	134,820 (45.8)	70,097 (45.7)	64,723 (45.8)	
3–4	29,601 (10.0)	15,511 (10.1)	14,090 (10.0)	
≥ 5	22,900 (7.8)	11,919 (7.8)	10,981 (7.8)	
Antenatal care, N (%)^2,3^	218,046	113,154	104,892	0.2955
≥ 4 ANC visits	134,446 (61.7)	69,784 (61.7)	64,662 (61.6)	
< 4 ANC visits	83,600 (38.3)	43,370 (38.3)	40,230 (38.4)	
Trimester of first ANC visit, N (%)^2,3^	259,453	135,254	124,199	0.0050
First (0–14 weeks)	170,639 (65.8)	88,475 (65.4)	82,164 (66.2)	
Second (15–28 weeks)	65,488 (25.2)	34,465 (25.5)	31,023 (25.0)	
Third (29–42 weeks)	23,326 (9.0)	12,314 (9.1)	11,012 (8.9)	
Complications of pregnancy, N (%)^2,3^				
Evidence of hypertensive disease/severe pre-eclampsia/eclampsia	10,072/297,108 (3.4)	5260/154,604 (3.4)	4812/142,504 (3.4)	0.4986
Severe antepartum hemorrhage	4341/297,198 (1.5)	2361/154,652 (1.5)	980/142,546 (1.4)	0.0022
BMI ≥ 25	21,660/291,335 (7.4)	11,313/151,628 (7.5)	10,347/139,707 (7.4)	0.5177
Hemoglobin measured at/before enrollment < 8 mg/dl	5762/212,960 (2.7)	2930/110,629 (2.6)	2832/102,331 (2.8)	0.1905
Hemoglobin measured after enrollment and before delivery < 8 mg/dl	2794/79,063 (3.5)	1424/40,865 (3.5)	1370/38,198 (3.6)	0.4455
Multiple birth	5297/297,444 (1.8)	2,704/154,748 (1.7)	2593/142,696 (1.8)	0.1330

^1^All MNH Registry 2010–2018 infants born in 08 Belagavi, 11 Nagpur, and 09 Pakistan with exclusions miscarriage, MTP, and maternal deaths prior to delivery

^2^In cases of multiple births, maternal demographic variables are counted more than once

^3^Categorical variable p-values are computed with a cluster-stratified CMH test

**Table 2:** Baseline demographic and clinical characteristics by site and infant gender

Characteristic	India		Pakistan		India p-value	Pakistan p-value
	Male	Female	Male	Female		
Infants^1^	107,148	99,006	47,642	43,713		
Maternal age, N (%)^2,3^	107,085	98,963	47,537	43,644	0.7659	0.0566
< 20	7454 (7.0)	6980 (7.1)	1871 (3.9)	1846 (4.2)		
20–35	99,304 (92.7)	91,681 (92.6)	43,036 (90.5)	39,414 (90.3)		
> 35	327 (0.3)	302 (0.3)	2630 (5.5)	2384 (5.5)		
Maternal education, N (%)^2,3^	106,764	98,687	47,542	43,606	0.6538	0.0739
No formal schooling	12,799 (12.0)	11,946 (12.1)	39,211 (82.5)	36,050 (82.7)		
Primary or secondary	80,598 (75.5)	74,326 (75.3)	7161 (15.1)	6418 (14.7)		
University+	13,367 (12.5)	12,415 (12.6)	170 (2.5)	1138 (2.6)		
Parity, N (%)^2,3^	106,860	98,757	46,450	42,588	0.5140	0.8840
0	46,925 (43.9)	43,478 (44.0)	8858 (19.1)	8073 (19.0)		
1–2	54,561 (51.1)	50,422 (51.1)	15,536 (33.4)	14,301 (33.6)		
3–4	4961 (4.6)	4482 (4.5)	10,550 (22.7)	9608 (22.6)		
≥ 5	413 (0.4)	375 (0.4)	11,506 (24.8)	10,606 (24.9)		
Antenatal care, N (%)^2,3^	76,938	71,450	36,216	33,442	0.8103	0.1722
≥ 4 ANC visits	57,335 (74.5)	53,282 (74.6)	12,449 (34.4)	11,380 (34.0)		
< 4 ANC visits	19,603 (25.5)	18,168 (25.4)	23,767 (65.6)	22,062 (66.0)		
Trimester of first ANC visit, N (%)^2,3^	100,917	93,130	34,337	31,069	0.0054	0.6086
First (0–14 wks)	76,072 (75.4)	70,764 (76.0)	12,403 (36.1)	11,400 (36.7)		
Second (15–28 wks)	20,809 (20.6)	18,758 (20.1)	13,656 (39.8)	12,265 (39.5)		
Third (29–42 wks)	4036 (4.0)	3608 (3.9)	8278 (24.1)	7404 (23.8)		
Complications of pregnancy, N (%)^2,3^						
Evidence of hypertensive disease/severe pre-eclampsia/eclampsia	2940/107,006 (2.7)	2600/98,823 (2.6)	2320/47,598 (4.9)	2212/43,681 (5.1)	0.0694	0.3032
Severe antepartum hemorrhage	621/107,051 (0.6)	546/98,862 (0.6)	1740/47,601 (3.7)	1434/43,684 (3.3)	0.4256	0.0019
BMI ≥ 25	5692/104,187 (5.5)	5151/96,163 (5.4)	5621/47,441 (11.8)	5196/43,544 (11.9)	0.2501	0.7869
Hemoglobin measured at/before enrollment < 8 mg/dl	1271/100,935 (1.3)	1229/93,184 (1.3)	1659/9,694 (17.1)	1603/9,147 (17.5)	0.2337	0.5020
Hemoglobin measured after enrollment and before delivery < 8 mg/dl	466/36,273 (1.3)	406/33,891 (1.2)	958/4,592 (20.9)	964/4,307 (22.4)	0.2871	0.0765
Multiple birth	1590/107,136 (1.5)	1587/99,001 (1.6)	1114/47,612 (2.3)	1006/43,695 (2.3)	0.0243	0.6984

^1^All MNH Registry 2010–2018 infants born in 08 Belagavi, 11 Nagpur, and 09 Pakistan with exclusions miscarriage, MTP, and maternal deaths prior to delivery

^2^In cases of multiple births, maternal demographic variables are counted more than once

^3^Categorical variable p-values are computed with a cluster-stratified CMH test, while continuous variable p-values are computed with a Wilcoxon rank-sum test

Male infants were more likely to be attended by a physician at birth (50.9% vs. 49.6%), born at a hospital (57.9% vs. 56.5%), delivered by cesarean (18.0% vs. 17.1%) and received bag and mask resuscitation immediately after birth (6.7% vs. 5.5%) (Table 3). There were clinically insignificant differences in mean birth weight (2780 ± 486 g vs. 2717 ± 466 g) and gestational age (38.3 ± 3.4 vs. 38.5 ± 3.4) between male and female infants. There was no significant difference in preterm births between male and female infants. Similar results were found in male and female infants at the Indian and Pakistani sites with an exception of a slightly higher preterm birth rate in males in India (11% vs. 10.6%) (Table 4).

**Table 3:** Delivery methods and outcomes by infant gender

Characteristic	Overall	Infant Gender		p-value
		Male	Female	
Infants^1^	297,509	154,790	142,719	
Birth attendant, N (%)^2^	297,462	154,766	142,696	< .0001
Physician	149,596 (50.3)	78,835 (50.9)	70,761 (49.6)	
Nurse/nurse midwife/LHW/HW	94,051 (31.6)	48,567 (31.4)	45,484 (31.9)	
TBA	43,296 (14.6)	22,066 (14.3)	21,230 (14.9)	
Family/self/other	10,519 (3.5)	5298 (3.4)	5221 (3.7)	
Delivery location, N (%)^2^	297,439	154,754	142,685	< .0001
Hospital	170,209 (57.2)	89,606 (57.9)	80,603 (56.5)	
Clinic/health center	82,577 (27.8)	42,592 (27.5)	39,985 (28.0)	
Home/other	44,653 (15.0)	22,556 (14.6)	22,097 (15.5)	
Delivery mode, N (%)^2^	297,503	154,788	142,715	< .0001
Vaginal	240,721 (80.9)	124,493 (80.4)	116,228 (81.4)	
Vaginal, assisted	4460 (1.5)	2389 (1.5)	2071 (1.5)	
C-section	52,322 (17.6)	27,906 (18.0)	24,416 (17.1)	
Bag and mask resuscitation, N (%)^2^	295,508	153,749	141,759	< .0001
Yes	18,177 (6.2)	10,353 (6.7)	7824 (5.5)	
No	277,331 (93.8)	143,396 (93.3)	133,935 (94.5)	
Birth weight (measured and estimated), N (%)^2^	297,022	154,514	142,508	< .0001
< 1000 g	1844 (0.6)	976 (0.6)	868 (0.6)	
1000–1499 g	4143 (1.4)	2172 (1.4)	1971 (1.4)	
1500–2499 g	49,117 (16.5)	23,619 (15.3)	25,498 (17.9)	
≥ 2500 g	241,918 (81.4)	127,747 (82.7)	114,171 (80.1)	
Birth weight (measured), N (%)^2^	292,082 (98.2)	151,798 (98.1)	140,284 (98.3)	< .0001
Mean (std)	2,749.6 (477.5)	2,779.8 (486.2)	2,716.9 (465.7)	
Median (25p-75p)	2,750.0 (2,500.0, 3,000.0)	2,800.0 (2,500.0, 3,000.0)	2,700.0 (2,500.0, 3,000.0)	
Gestational age, N (%)^2^	289,018	150,474	138,544	0.3232
Preterm	39,302 (13.6)	20,547 (13.7)	18,755 (13.5)	
Term	249,716 (86.4)	129,927 (86.3)	119,789 (86.5)	
Gestational age at delivery, N (%)^2^	280,287 (94.2)	145,633 (94.1)	134,654 (94.3)	< .0001
Mean (std)	38.4 (3.4)	38.3 (3.4)	38.5 (3.4)	
Median (25p-75p)	39.0 (37.0, 40.0)	39.0 (37.0, 40.0)	39.0 (37.0, 40.0)	

^1^All MNH Registry 2010–2018 infants born in 08 Belagavi, 11 Nagpur, and 09 Pakistan with exclusions miscarriage, MTP, and maternal deaths prior to delivery

^2^Categorical variable p-values are computed with a cluster-stratified CMH test, while continuous variable p-values are computed with a Wilcoxon rank-sum test

**Table 4:** Delivery methods and outcomes by site and infant gender

	India		Pakistan		India p-value	Pakistan p-value
Characteristic	Male	Female	Male	Female		
Infants^1^	107,148	99,006	47,642	43,713		
Birth attendant, N (%)^2^	107,145	99,001	47,621	43,695	< .0001	< .0001
Physician	65,439 (61.1)	59,167 (59.8)	13,396 (28.1)	11,594 (26.5)		
Nurse/nurse midwife/LHW/HW	38,210 (35.7)	36,170 (36.5)	10,357 (21.7)	9314 (21.3)		
TBA	1616 (1.5)	1626 (1.6)	20,450 (42.9)	19,604 (44.9)		
Family/self/other	1880 (1.8)	2038 (2.1)	3418 (7.2)	3183 (7.3)		
Delivery location, N (%)^2^	107,127	98,983	47,627	43,702	< .0001	< .0001
Hospital	73,481 (68.6)	66,401 (67.1)	16,125 (33.9)	14,202 (32.5)		
Clinic/health center	29,562 (27.6)	28,352 (28.6)	13,030 (27.4)	11,633 (26.6)		
Home/other	4084 (3.8)	4230 (4.3)	18,472 (38.8)	17,867 (40.9)		
Delivery mode, N (%)^2^	107,147	99,003	47,641	43,712	< .0001	< .0001
Vaginal	84,142 (78.5)	78,743 (79.5)	40,351 (84.7)	37,485 (85.8)		
Vaginal, assisted	760 (0.7)	666 (0.7)	1629 (3.4)	1405 (3.2)		
C-section	22,245 (20.8)	19,594 (19.8)	5661 (11.9)	4822 (11.0)		
Bag and mask resuscitation, N (%)^2^	106,283	98,185	47,466	43,574	< .0001	< .0001
Yes	4952 (4.7)	3,888 (4.0)	5401 (11.4)	3936 (9.0)		
No	101,331 (95.3)	94,297 (96.0)	42,065 (88.6)	39,638 (91.0)		
Birth weight (measured and estimated), N (%)^2^	107,003	98,898	47,511	43,610	< .0001	< .0001
< 1000 g	639 (0.6)	585 (0.6)	337 (0.7)	283 (0.6)		
1000—1499 g	1258 (1.2)	1159 (1.2)	914 (1.9)	812 (1.9)		
1500—2499 g	15,629 (14.6)	16,931 (17.1)	7990 (16.8)	8567 (19.6)		
≥ 2500 g	89,477 (83.6)	80,223 (81.1)	38,270 (80.5)	33,948 (77.8)		
Birth weight (measured), N (%)^2^	106,429 (99.3)	98,370 (99.4)	45,369 (95.2)	41,914 (95.9)	< .0001	< .0001
Mean (std)	2747.3 (453.8)	2687.4 (431.5)	2856.0 (547.4)	2786.2 (531.3)		
Median (25p-75p)	2750.0 (2500.0, 3000.0)	2700.0 (2500.0, 3000.0)	2900.0 (2,510.0, 3150.0)	2800.0 (2500.0, 3050.0)		
Gestational age, N (%)^2^	104,865	96,750	45,609	41,794	0.0056	0.0799
Preterm	11,559 (11.0)	10,297 (10.6)	8988 (19.7)	8458 (20.2)		
Term	93,306 (89.0)	86,453 (89.4)	36,621 (80.3)	33,336 (79.8)		
Gestational age at delivery, N (%)^2^	102,366 (95.5)	94,763 (95.7)	43,267 (90.8)	39,891 (91.3)	< .0001	< .0001
Mean (std)	38.6 (3.1)	38.8 (3.0)	37.7 (4.1)	37.8 (4.0)		
Median (25p-75p)	39.0 (38.0, 40.0)	39.0 (38.0, 40.0)	38.0 (36.0, 40.0)	38.0 (36.0, 40.0)		

^1^All MNH Registry 2010–2018 infants born in 08 Belagavi, 11 Nagpur, and 09 Pakistan with exclusions miscarriage, MTP, and maternal deaths prior to delivery

^2^Categorical variable p-values are computed with a cluster-stratified CMH test, while continuous variable p-values are computed with a Wilcoxon rank-sum test

The rate of stillbirths was significantly higher in male infants compared to females (Table 5). A total of 9309 infants (32.3/1,000 live births) died between 1 and 42 days 7033 infants (75.6%) died between 1 and 7 days and 2276 infants (24.4%) died between 8 and 42 days. Early neonatal mortality was significantly higher in male infants compared to female infants. However, there were no significant differences in late neonatal mortality and mortality between 29 and 42 days between the two groups. More male infants were hospitalized within 42 days after birth compared to female infants. Similar patterns of mortality rates between the two groups were observed when the Indian and Pakistani sites were analyzed separately (Table 6). However, the rates of mortality were much higher in the Pakistani site compared to the India sites.

**Table 5:** Death and hospitalization after delivery by infant gender

Characteristic	Overall	Infant gender		Unadjusted RR	p-value
		Male	Female		
Stillbirths, N (rate/1000)^1,2,3^	8648 (29.1)	4806 (31.0)	3842 (26.9)	1.15 (1.10, 1.20)	< .0001
Perinatal mortality rates, N (rate/1000)^1,2,3^					
≤ 7 days	15,681 (52.9)	8854 (57.4)	6827 (48.0)	1.19 (1.15, 1.23)	< .0001
≤ 28 days	17,398 (58.7)	767 (63.3)	631 (53.6)	1.17 (1.14, 1.21)	< .0001
Neonatal mortality rates, N (rate/1000)^1,2,3^					
1–28 days	8750 (30.4)	4961 (33.2)	3789 (27.4)	1.21 (1.16, 1.26)	< .0001
1–7 days	7033 (24.4)	4048 (27.1)	2985 (21.6)	1.25 (1.19, 1.31)	< .0001
8–28 days	1717 (6.1)	913 (6.3)	804 (5.9)	1.06 (0.97, 1.15)	0.1760
29–42 days	559 (2.0)	303 (2.1)	256 (1.9)	1.11 (0.95, 1.29)	0.1946
Conditions requiring hospitalization in follow up, N (%)^1,2^	4074 (1.5)	2399 (1.8)	1675 (1.3)		< .0001

^1^All MNH Registry 2010–2018 infants born in 08 Belagavi, 11 Nagpur, and 09 Pakistan with exclusions miscarriage, MTP, and maternal deaths prior to delivery

^2^Categorical variable p-values are computed with a cluster-stratified CMH test

^3^Relative risks are unadjusted, calculated from a model using GEE to adjust for cluster

**Table 6:** Death and hospitalization after delivery by site and infant gender

Characteristic	India		Pakistan		Unadj. RR		p-value	
	Male	Female	Male	Female	India RR	Pakistan RR	Ind	Pak
Stillbirths, N (rate/1000)^1,2,3^	2444 (22.8)	2006 (20.3)	2362 (49.6)	1836 (42.0)	1.13 (1.05, 1.20)	1.18 (1.12, 1.24)	0.0006	< .0001
Perinatal mortality rates, N (rate/1000)^1,2,3^								
≤ 7 days	4558 (42.6)	3516 (35.5)	4296 (90.9)	3311 (76.3)	1.20 (1.14, 1.26)	1.19 (1.14, 1.25)	< .0001	< .0001
≤ 28 days	5025 (46.9)	3909 (39.5)	4742 (100.3)	3722 (85.8)	1.19 (1.14, 1.24)	1.17 (1.12, 1.22)	< .0001	< .0001
Neonatal mortality rates, N (rate/1000)^1,2,3^								
1–28 days	2581 (24.7)	1903 (19.6)	2,380 (53.0)	1886 (45.4)	1.26 (1.18, 1.34)	1.17 (1.10, 1.24)	< .0001	< .0001
1–7 days	2114 (20.2)	1510 (15.6)	1934 (43.1)	1475 (35.5)	1.30 (1.21, 1.39)	1.21 (1.14, 1.30)	< .0001	< .0001
8–28 days	467 (4.6)	393 (4.1)	446 (10.4)	411 (10.3)	1.11 (0.97, 1.26)	1.01 (0.91, 1.13)	0.1314	0.8068
29–42 days	122 (1.2)	112 (1.2)	181 (4.3)	144 (3.6)	1.02 (0.80, 1.31)	1.17 (0.95, 1.45)	0.8602	0.1367
Conditions requiring hospitalization in follow up, N (%)^1,2^	1716 (1.8)	1278 (1.4)	683 (1.7)	397 (1.1)			< .0001	< .0001

^1^All MNH Registry 2010–2018 infants born in 08 Belagavi, 11 Nagpur, and 09 Pakistan with exclusions miscarriage, MTP, and maternal deaths prior to delivery

^2^Categorical variable p-values are computed with a cluster-stratified CMH test

^3^Relative risks are unadjusted, calculated from a model using GEE to adjust for cluster

The probable causes of stillbirths and infant deaths between 1 and 42 days were similar in the male and female infants (Tables 7 and 8). Low birth weight/prematurity and birth asphyxia were the common probable causes for stillbirth as well as death between 1 and 42 days of age.

**Table 7:** Causes of death by infant gender

Characteristic	Overall	Infant Gender		p-value
		Male	Female	
Stillbirth cause of death, N (%)^1,2,3^	4091	2286	1805	0.5673
Birth asphyxia	495 (12.1)	294 (12.9)	201 (11.1)	
Low birth weight (< 2500 g)/prematurity	1454 (35.5)	803 (35.1)	651 (36.1)	
Infection	279 (6.8)	151 (6.6)	128 (7.1)	
Malformation	222 (5.4)	127 (5.6)	95 (5.3)	
Other	1641 (40.1)	911 (39.9)	730 (40.4)	
Neonatal death < 42 days cause of death, N (%)^1,2,3^	9128	5161	3967	0.2396
Birth asphyxia	2732 (29.9)	1,552 (30.1)	1180 (29.7)	
Low birth weight (< 2500 g)/prematurity	2850 (31.2)	1580 (30.6)	1270 (32.0)	
Infection/sepsis	986 (10.8)	548 (10.6)	438 (11.0)	
Congenital abnormalities/Malformation	454 (5.0)	251 (4.9)	203 (5.1)	
Other/no cause assigned	2106 (23.1)	1230 (23.8)	876 (22.1)	

^1^All MNH Registry 2010–2018 infants born in 08 Belagavi, 11 Nagpur, and 09 Pakistan with exclusions miscarriage, MTP, and maternal deaths prior to delivery

^2^Categorical variable p-values are computed with a cluster-stratified CMH test

^3^Physician-assigned stillbirth cause of death is only provided for Versions 0.1–1.4. Physician-assigned neonatal cause of death is constructed from all versions

**Table 8:** Infant causes of death by site and infant gender

Characteristic	India		Pakistan		India p-value	Pakistan p-value
	Male	Female	Male	Female		
Stillbirth cause of death, N (%)^1,2,3^	1224	1002	1062	803	0.3590	0.9886
Birth asphyxia	226 (18.5)	152 (15.2)	68 (6.4)	49 (6.1)		
Low birth weight (< 2500 g)/prematurity	461 (37.7)	395 (39.4)	342 (32.2)	256 (31.9)		
Infection	122 (10.0)	104 (10.4)	29 (2.7)	24 (3.0)		
Malformation	99 (8.1)	75 (7.5)	28 (2.6)	20 (2.5)		
Other	316 (25.8)	276 (27.5)	595 (56.0)	454 (56.5)		
Neonatal death < 42 days cause of death, N (%)^1,2,3^	2645	1967	2516	2000	0.3335	0.1421
Birth asphyxia	814 (30.8)	605 (30.8)	738 (29.3)	575 (28.8)		
Low birth weight (< 2500 g)/prematurity	901 (34.1)	685 (34.8)	679 (27.0)	585 (29.3)		
Infection/sepsis	216 (8.2)	188 (9.6)	332 (13.2)	250 (12.5)		
Congenital abnormalities/malformation	167 (6.3)	117 (5.9)	84 (3.3)	86 (4.3)		
Other/no cause assigned	547 (20.7)	372 (18.9)	683 (27.1)	504 (25.2)		

^1^All MNH Registry 2010–2018 infants born in 08 Belagavi, 11 Nagpur, and 09 Pakistan with exclusions miscarriage, MTP, and maternal deaths prior to delivery

^2^Categorical variable p-values are computed with a cluster-stratified CMH test

^3^Physician-assigned stillbirth cause of death is only provided for Versions 0.1–1.4. Physician-assigned neonatal cause of death is constructed from all versions

## Discussion

Over the past several decades, gender differences have been reported in stillbirth, neonatal and infant mortality with a higher incidence of death in male subjects [1–4]. However, several studies from south Asia have reported higher late neonatal and infant mortality rates in female subjects [10–12]. Our large population-based study involving the Global Network research sites in India and Pakistan demonstrated that more male infants were born compared to females. The rates of stillbirths and early neonatal mortality were significantly higher in male infants. The late neonatal mortality rate (8–28 days) and the mortality rate between ages 29–42 days were similar among the genders. Our analysis also shows the rates of stillbirth, early and late neonatal mortality and mortality between 29 and 42 days are higher for the Pakistani site compared to the two Indian sites.

The sex ratio at birth (ratio of male to female) in humans is reported to be slightly higher for males, with 1050 male births for every 1000 female births [20]. We report a higher sex ratio of 1080 males for every 1000 females at birth in our cohort. As mortality in males is higher throughout the life span, the ratio normalizes over time. Globally, the sex ratio at birth is reported at 1068:1000, with a regional variation from 1032:1000 in sub-Saharan Africa to 1133:000 in eastern Asia [20]. The sex ratio at birth from southern Asia was 1,086:1,000; similar to our cohort [20]. Gender preference and sex selective abortion, a common practice in south Asia is one likely cause for the higher male sex ratio at birth [21]. Despite enactment of laws prohibiting prenatal sex determination, sex-selected abortion is still performed in India, where the desire for small families and ideal sex composition led to a substantial increase in selective abortion of girls, especially after a first-born girl [22]. A more aggressive enforcement of laws prohibiting prenatal sex determination and sex-selected abortion may improve the sex ratio at birth.

The neonatal mortality rate is significantly higher among male infants in high-income countries [1–4]. Our data confirm higher neonatal mortality rates in male infants. A recent study from northern India also reported a similar trend with a higher neonatal mortality in male infants [10]. Our data also indicate that the rate of stillbirths and perinatal mortality is higher in males compared to females. Mondal et al. in a meta-analysis including more than 30 million births worldwide, reported a 10% higher risk of stillbirths in males [23]. However, in a recent study of mixed-gender twins, Zhao et al. did not find a significant gender difference in the rate of stillbirths [24]. Several explanations for increased perinatal and neonatal mortality observed in males have been proposed. The risks of intrauterine growth restriction, prematurity, respiratory distress syndrome, and birth asphyxia are higher in male infants [25–27]. Boys are also heavier than girls at birth, which can contribute to higher rates of delivery complications and birth injuries [5, 12]. In our cohort, the rate of prematurity was similar in the two groups and birth weights were lower in females, suggesting prematurity and intrauterine growth restriction may not have contributed to higher mortality in males. More male infants were provided bag and mask ventilation at birth but birth asphyxia as a cause of death was similar in the two groups. Respiratory distress syndrome and respiratory morbidities are reported to be more common in male neonates [28–30]. Unfortunately, we do not have data on respiratory morbidities in our cohort. The underlying mechanisms and cause for increased mortality due to respiratory illness in our study population cannot be determined.

Our results highlight the importance of analyzing data by stratifying neonatal mortality into early neonatal (1–7 days), late neonatal mortalities (8–28) and mortality between 29–42 days. Our data on neonatal mortality indicates higher death rates among male infants. However, the neonatal mortality is higher in male infants due to increased deaths during the first week of life after which the survival rates are similar in male and female infants. Female infants’ survival rates in high-income nations are higher than male infants [5, 24]. However, several studies from South Asia have reported a higher mortality in female infants after 7 days of age [10, 12, 13]. Rosentock et al. in a study from Nepal, reported that boys are at 20% greater risk of early neonatal mortality (< 7 days) and girls have a 43% greater risk of mortality during the late neonatal period (8–28 days) [12]. In a similar study from urban Pakistan, late neonatal mortality (8–28 days) was higher in females, compared to male infants (19.5/1000 vs. 5.5/1000) [13]. In a large database study from rural northern India, the infant mortality rate was significantly higher in females compared to males (7.2% vs. 6.3%) [10]. The death rate was higher in females during the post neonatal period (> 28 days) but not during the neonatal period. However, in our cohort the late neonatal mortality rate (after 7 days) was not higher in female infants compared to males. In India, parents provide less household expenditures for seeking healthcare for female infants compared to male infants [14, 15]. Gupta et al*.*, reported a strong bias against females for seeking medical attention both for outpatient and inpatient care [31]. A recent study from a northern state in India reported that the girls receive less care than boys for neonatal illness [32]. It is possible that a prospective follow-up at 6 weeks for all infants including girls in our cohort may have contributed in reducing late neonatal mortality in females by providing healthcare access. Despite biological advantages and better survival of female infants in high-income nations, the risk of neonatal mortality after 7 days of life among girls is similar to boys in our cohort. We speculate that biological and genetic advantages in females were offset by societal factors that limit resources for care of female infants. The rate of hospitalization after birth was higher in male infants in our population. This suggests that either male infants were more likely to become ill after birth or there was a preferential health-seeking behavior for male infants. Educating parents on the importance of providing equal resources to male and female children and encouraging them to seek preventive and acute care for infants of both sexes may improve mortality after 7 days in girls.

Our study has several strengths; data were analyzed from a large population-based registry in which data are prospectively collected on maternal and infant demographic and clinical characteristics and outcomes. Common protocols and methodologies were used across the sites to document the pregnancy and neonatal outcomes, including stillbirths and neonatal deaths. Longitudinal follow up in this large cohort from early pregnancy to six weeks post-partum allowed for assessment and control of many antenatal factors, and evaluation of trends in early and late neonatal deaths as well as hospitalization after delivery. We also recognize some important limitations of this study. The data were collected from limited geographical areas in India and Pakistan, and the results may not reflect the trends over all for south Asia. There were significant statistical differences in several variables in the baseline demographic, clinical characteristics, and delivery methods in the two groups. We believe that the significant differences were due to a large sample size and may not be clinically significant. We were unable to determine the underlying mechanisms and cause for increased perinatal mortality in males and the loss of survival advantage in females after 7 days of age.

## Conclusion

In summary, our data indicate that the risks of stillbirths and early neonatal mortality are higher among male infants than females. However, there were no gender differences in late neonatal and mortality between 29 and 42 days of age. Our results highlight the importance of stratifying neonatal mortality into early and late neonatal periods to better understand the impact of gender on neonatal mortality. We speculate that genetic and biological advantages associated with the female gender do not reduce the late neonatal mortality in girls, or these advantages are possibly offset by gender preference and preferential health-care seeking behavior. Our data contribute to the body of evidence suggesting that fetal sex is an important risk factor for stillbirths and early neonatal mortality. The absence of a survival advantage among female infants after the early neonatal period needs further study in resource-limited countries. The information from this study will help to develop strategies and identify measures that may help to reduce sex-specific mortality.

## Data Availability

Not applicable.

## Abbreviations

BMI: Body mass index
GN: Global Network, Global Network for Women’s and Children’s Health Research
MNHR: Maternal Newborn Health Registry
NICHD: National Institute of Child Health and Human Development
RA: Registry Administrator
